# The prevalence of IgE-dependent food allergy in asthmatic children

**DOI:** 10.1186/2045-7022-3-S3-P61

**Published:** 2013-07-25

**Authors:** A Krogulska, J Białek, M Funkowicz, K Wąsowska-Królikowska

**Affiliations:** 1 Department of Pediatric Allergology, Gastroenterology and Nutrition, Medical University Łódź, Łódź, Poland

## Background

Food allergy is rarely considered a trigger for asthma. However, the each part of respiratory tract may be an effector organ of allergic reaction provoked by food. The aim of this study was to evaluate the prevalence of IgE-dependent food allergy in asthmatic children.

## Methods

The initial study group consisted of 506 children with atopic asthma, aged 6-18 years. Methodology included: history, physical examination, pulmonary function tests, skin prick tests (SPT), specific IgE using the UniCAP 100 Pharmacia Upjohn and oral challenge tests, which were performed using the DBPCFC method.

## Results

Based on a survey showed that 180/362 (49.7%) children with asthma had symptoms that were associated with the consumption of food. Sensitization to food allergens was found in 70 (19.3%) children with asthma. Oral provocation tests using DBPCFC was performed in 50 children with asthma, in whom the results of the interview and / or SPT and / or specific IgE indicated a possible involvement of food allergens on symptoms of IgE-mediated reactions. The positive results of oral food tests was found in 20 (5.5%) children with asthma. After including 4 children with a well-documented history of anaphylactic reactions, IgE-mediated food allergy was diagnosed in 24 patients with asthma (6.6%). Respiratory symptoms as a result of oral food challenges occurred in 3% of children with asthma, however the asthmatic symptoms related to 9/362 (2.5%) of the children.

## Conclusion

IgE-dependent food allergy was diagnosed in 6.6% of children with asthma. Respiratory symptoms as a result of food allergen provocation occurred in 3% of children with asthma.

## Disclosure of interest

None declared.
